# Supplementary material for: Variable Legionella Response to Building Occupancy Patterns and Precautionary Flushing
Source: Microorganisms. 2022 Mar 3;10(3):555. doi: 10.3390/microorganisms10030555 (PMC8950775; doi:10.3390/microorganisms10030555)
Supplement: Supplementary file 1 [file microorganisms-10-00555-s001.zip › microorganisms-1604111-supplementary.pdf]

Supplementary Information for Variable *Legionella* Response to Building Occupancy Patterns and Precautionary Flushing

William J Rhoads[1], Meril Sindelar[1,2], Céline Margot[1,3], Nadine Graf[1,3], Frederik Hammes[1]

[1]Department of Environmental Microbiology, Eawag: Swiss Federal Institute of Aquatic Science and Technology, Dübendorf, Switzerland

[2] Department of Biology, ETH Zürich, Zürich, Switzerland

[3] Department of Environmental Systems Science, Institute of Biogeochemistry and Pollutant Dynamics, ETH Zürich, Zürich, Switzerland

Author Contact: william.rhoads@eawag.ch

Table of Contents

Table S1. Date and number of samples by type collected for each data collection period in the case study 3

Table S2. Number of samples of each type (Regular and Random) during each sampling of the recommissioning data collection period 4

Table S3. Primer/probe sequences, ddPCR reaction master mix recipe per sample, and thermocycler settings 4

Table S4. Location of Data loggers for each of the full-scale study periods 4

Table S5. 25th, 50th, 75th, and average temperatures for the B-floor (hot water supply and hot water return locations nearest to boiler) for a 24-hour period (00:00-23:59) and during business hours (08:00-15:00) (typical) 5

Figure S1. General building layout and overview of hot water plumbing design 6

Figure S2. DNA Extraction process control chart (gene copies/µL PCR reaction) 7

Figure S3. ddPCR Positive Control process control chart (gene copies/µL PCR reaction) 8

Figure S4. *Legionella* spp and *L. pneumophila* ddPCR assay linearity 9

Figure S5. Determination of the limit of quantification (LOQ) 9

Figure S6. Determination of the limit of detection (LOD) 10

Figure S7. Pipe surface temperature from a typical mid-week period during the lockdown phase of data collection 10

Figure S8. A) Total cell counts, B) intact cell counts, and C) percent of intact cells (relative to total cells) in first draw samples (after wasting the first 50 mL) on Floor C (green) and Floor E (yellow) during the 4-week Controlled Floor Stagnation 11

Figure S9. Empirical cumulative distribution function of the hot water supply and return on the closest floor to the boiler (B Floor) on a day the boiler set point was A) 45 °C and B) 60 °C 11

Figure S10. Pipe surface temperatures of the hot water recirculation supply and return pipes (left) and location where temperature sensors were installed (right). 12

Figure S11. Empirical cumulative distribution function of the hot water supply and return on the floor closest to the boiler (B Floor) from 1 week of data 13

Figure S12. Pipe surface temperature from a typical mid-week period during the lockdown phase of data collection. 14

Figure S13. *L. pneumophila* culture numbers from immediately after the COVID Lockdown and during the Recommissioning phase of data collection between Repeatedly and Randomly sampled outlets for A) first draw samples and B) 5-minute flushed samples. 15

Figure S14. Water temperature at the boiler outlet ("Top of Boiler") and bottom of boiler during the recommissioning flushing activities on April 30, 2020. Recommissioning flushing began around 08:30 and ended around 16:00. 16

Figure S15. Total and intact cell counts for randomly compared to repeatedly sampled outlets in first draw samples from outlets served by the floor loops (panel A and B) and risers (panel C and D). 17

Figure S16. Total and intact cell counts for randomly compared to repeatedly sampled outlets in 5-minute flushed samples from outlets served by the floor loops (panel A and B) and risers (panel C and D). 18

Figure S17. *L. pneumophila* culture number trends with A) total cell counts, B) intact cell counts, C) percent of intact cell counts, and D) sample temperature. No discernable trends were identified. 19

Table S1. Date and number of samples by type collected for each data collection period in the case study

Period	Date	First Draw	5-min Flush	Total
Routine	1/4/2019	16	12	28
	1/13/2019	5	5	10
	1/20/2019	6	4	10
	1/27/2019	7	3	10
	3/15/2019	6	2	8
	5/6/2019	7	4	11
	7/1/2019	7	3	10
	10/18/2019	9	3	12
	12/13/2019	2	1	3
After Winter Break 2019	1/27/2020	9	3	12
	2/24/2020	7	3	10
COVID Lockdown	4/29/2020	40	19	59
Recommissioning	5/6/2020	19	9	28
	5/11/2020	19	9	28
	5/18/2020	19	9	28
	5/25/2020	15	13	28
	6/1/2020	15	13	28
	6/8/2020	15	13	28
	7/27/2020	12	2	14
	10/30/2020	6	6	12
Winter Break 2020	12/21/2020	36	14	50
	1/25/2021	36	14	50
Slow Boiler Flushing	4/14/2021	8	8	16
	4/21/2021	7	7	14
	4/28/2021	7	6	13
	5/3/2021	8	8	16
	5/10/2021	8	8	16
Fast Boiler Flushing	5/17/2021	8	8	16
	5/24/2021	7	6	13
	6/2/2021	7	7	14
	6/16/2021	7	6	13

Table S2. Number of samples of each type (Regular and Random) during each sampling of the recommissioning data collection period

Location	Regular			Random		
	First Draw	5-min Flush	Total$_{Repeat}$	First Draw	5-min Flush	Total$_{Random}$
Office Riser	1	1	2	0	0	0
Lab Riser	4	4	8	0	0	0
Lab Floor Loop	5	3	8	6-8	0-4	varies
**Total (n=28)**	10	8	**18**	varies	varies	**8**

Table S3. Primer/probe sequences, ddPCR reaction master mix recipe per sample, and thermocycler settings

**Primer/Probe**	**Sequence**
ssrA forward	GGC GAC CTG GCT TC
ssrA reverse	TCA TCG TTT GCA TTT ATA TTT A
ssrA probe	HEX-ACG TGG GTT GCA A-BHQ1
mip forward	TTG TCT TAT AGC ATT GGT GCC G
mip reverse	CCA ATT GAG CGC CAC TCA TAG
mip probe	FAM-CGG AAG CAA TGG CTA AAG GCA TGC A-BHQ1

**Component**	**Final Conc**	**Sample (uL)**
RNAse Free Water	-	10.53
PerfeCTa Multiplex ToughMix 5x	1x	5.4
Fluorescein 1uM	100 nM	2.7
Forward Primer 1 (20uM)	0.6 µM	0.675
Reverse Primer 1 (20uM)	0.6 µM	0.675
Probe 1 (20uM)	0.15 µM	0.135
Forward Primer 2 (20uM)	0.4 µM	0.675
Reverse Primer 2 (20uM)	0.4 µM	0.675
Probe 2 (20uM)	0.15 µM	0.135
DNA Template	-	5.4
Total	**-**	***27***

**Cylce Step**	**Settings**
Partitioning	12 min / 40°C
Enzyme activation	10 min / 95°C
45 cycles	15 sec / 95°C; 60 sec / 55 °C
Depressurization	Ambient Temperature

Table S4. Location of Data loggers for each of the full-scale study periods

**Period**	**Beginning and End of Floor Loops**	
COVID-Lockdown	B	B-G
Recommissioning	C, E, G	
Controlled Floor Stagnation	C, E	C71, C76; E70, E77

Table S5. 25th, 50th, 75th, and average temperatures for the B-floor (hot water supply and hot water return locations nearest to boiler) for a 24-hour period (00:00-23:59) and during business hours (08:00-15:00) (typical)

Parameter	45 °C Day				60 °C Day			
	Supply		Return		Supply		Return	
	00:00-23:59	08:00-15:00	00:00-23:59	08:00-15:00	00:00-23:59	08:00-15:00	00:00-23:59	08:00-15:00
25th %ile	30.4	42.3	26	37.3	55.6	55.4	46.6	46.8
50th %ile	42.1	42.8	36.8	37.6	56.4	55.9	47.2	47.2
75th %ile	43	43.6	37.5	37.7	57.3	56.4	47.4	47.4
Average	37.4	43	32.5	37.5	55.3	55.8	45.6	47.4

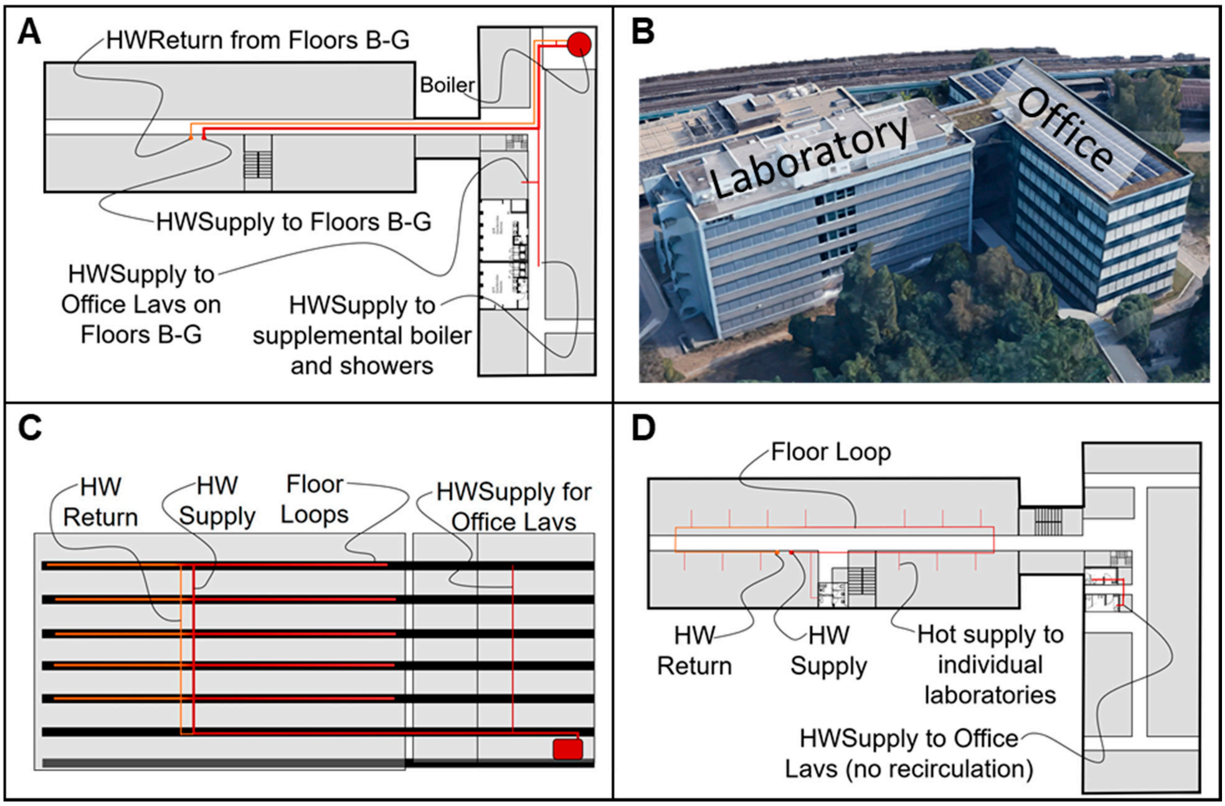

Figure S1. General building layout and overview of hot water plumbing design.

A) Plan view of Floor A where the boiler is located in the basement of the office building. Hot water supply is piped to a vertical riser for the office and laboratory building public outlets (e.g., lavatories, kitchettes), but is recirculated in the laboratory building to supply laboratories on each floor. B) An aerial photo of the Eawag research buildings. The laboratory and office buildings are labelled. C) Profile view of Eawag research buildings. The hot water is supplied in one vertical riser in the office and laboratory buildings to public spaces. Hot water is supplied and returned in one vertical recirculating riser in the laboratory building and distributed to outlets through passively recirculated floor loops. D) Plan view of Floors B-H (typical).

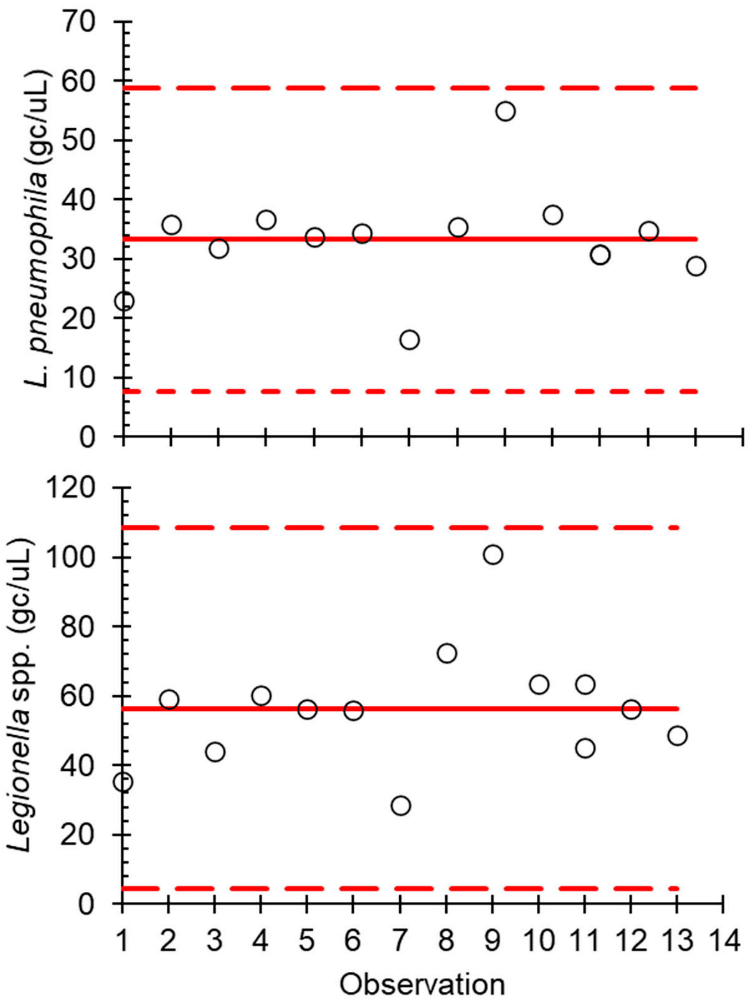

Figure S2. DNA Extraction process control chart (gene copies/µL PCR reaction)

(top) *L. pneumophila* and (bottom) *Legionella* spp. DNA extraction positive controls were created from a reactor system naturally colonized with *Legionella*. The replicate controls were made by filter concentration 100 mL of reactor water through the same 0.2 um polycarbonate filter used in the study. To do this 2 L of water from the reactor was collected, mixed, and 100 mL aliquots filtered. The solid red line is the average of all DNA extraction positive controls and the red dashed lines are 3X the standard deviation.

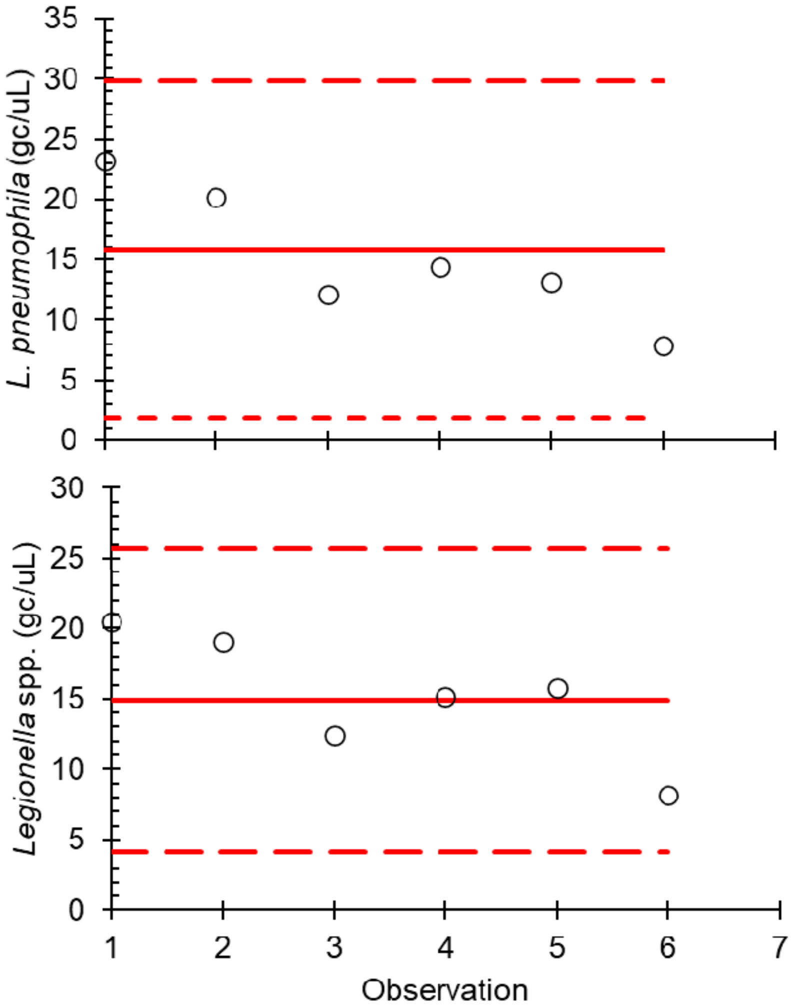

Figure S3. ddPCR Positive Control process control chart (gene copies/µL PCR reaction)

(top) *L. pneumophila* and (bottom) *Legionella* spp.ddPCR positive control results. 100 gc per rection (20 gc/uL as plotted above) were loaded into each thermocycler run. The solid red line is the average of all DNA extraction positive controls and the red dashed lines are 3X the standard deviation.

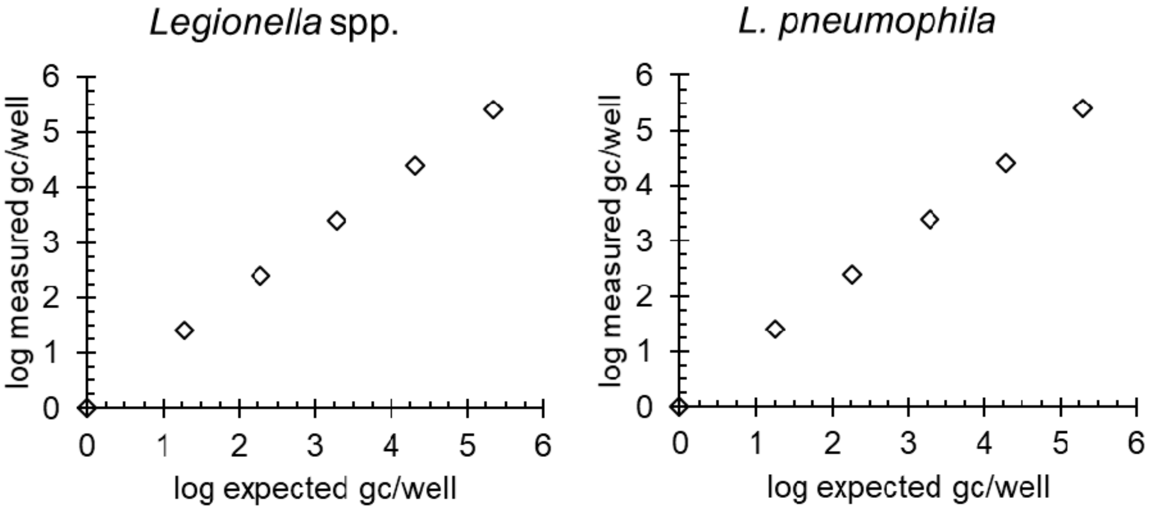

Figure S4. *Legionella* spp and *L. pneumophila* ddPCR assay linearity.

Expected gc/well was determined according to manufacture specifications (Centre National de Référence des Légionelles)

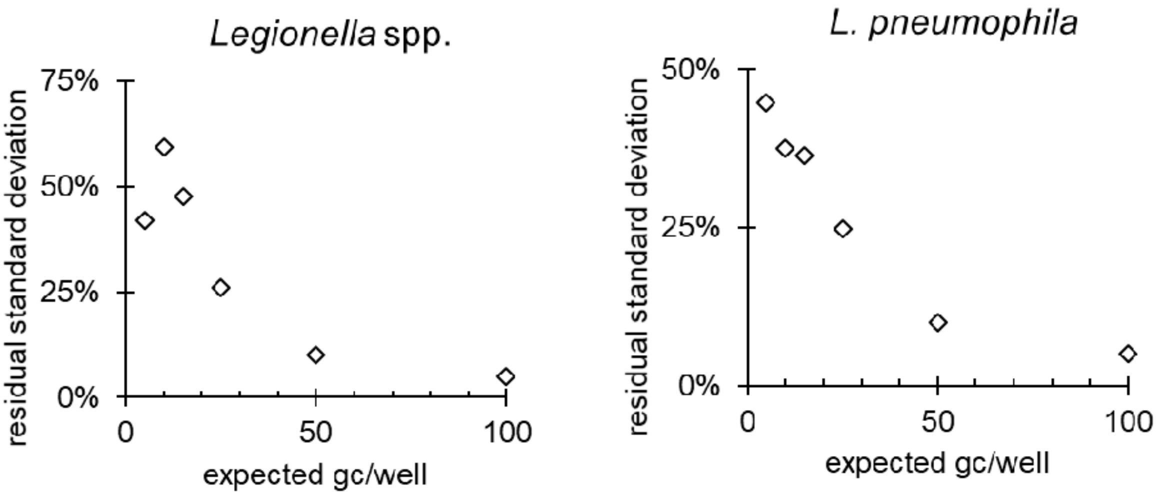

Figure S5. Determination of the limit of quantification (LOQ)

25 gc/well had a residual standard deviation of <= 25%

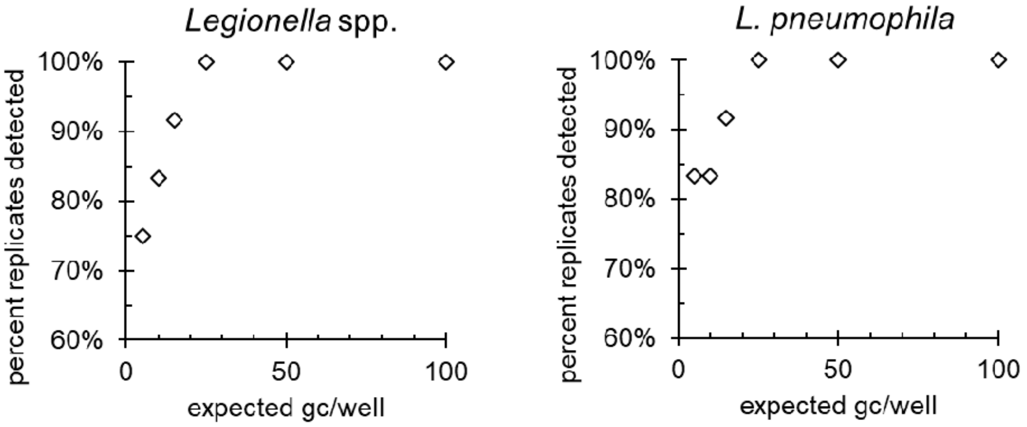

Figure S6. Determination of the limit of detection (LOD)

12 gc/well was detected in at least 90% of replicates

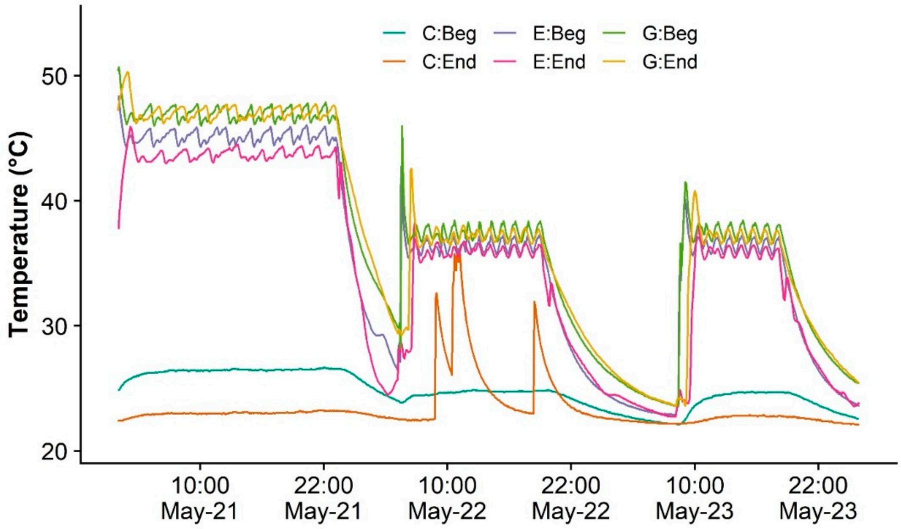

Figure S7. Pipe surface temperature from a typical mid-week period during the lockdown phase of data collection.

May 21, 2020 was a 60 °C boiler operation day; May 22-23, 2020 was a 45 °C boiler operation day. Data was collected at the inlet and outlet of the passive recirculating loops for Floors C, E, and G. Floor C was characterized by near ambient temperatures due to a closed ball valve in the middle of the floor loop that prevented passive circulation. Floors E and G demonstrate slightly different recirculating capacity through their median temperatures during business hours while the pump was on. May 22, 2020 demonstrates several hot water demands on Floor C.

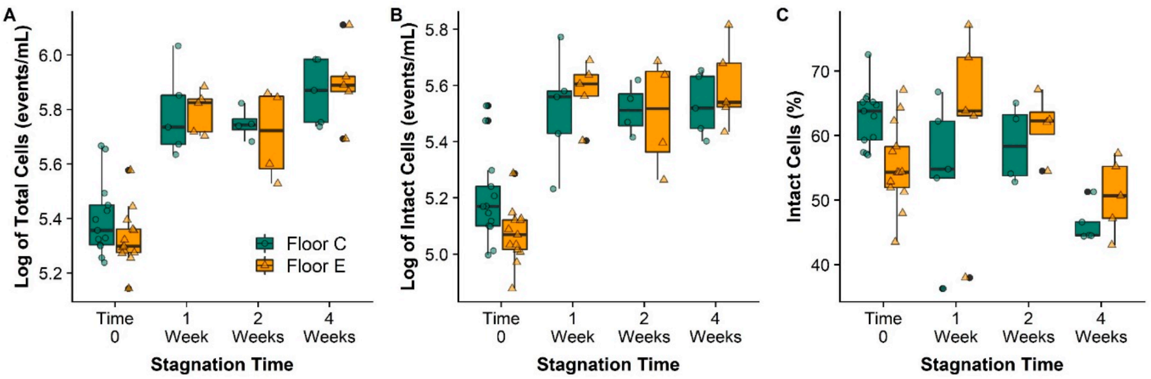

Figure S8. A) Total cell counts, B) intact cell counts, and C) percent of intact cells (relative to total cells) in first draw samples (after wasting the first 50 mL) on Floor C (green) and Floor E (yellow) during the 4-week Controlled Floor Stagnation.

Floors C and E were chosen because they exhibited characteristically good and poor passive recirculation, respectively. The majority of bacterial regrowth (TCC and ICC) occurred in the first week and then growth rate leveled off. Some evidence of cell accumulation was observed in TCC after 2 and 4 weeks of stagnation. The percent of intact cells generally decreased as stagnation time increased, as expected without exchange of nutrients or removal of dead/injured cells. There were no differences in TCC or ICC individually between the floors; however, the percent of intact cells was consistently higher in outlets on Floor E, which had good recirculation. Passive recirculation with the floor loop may have delivered nutrients to the outlets on Floor E.

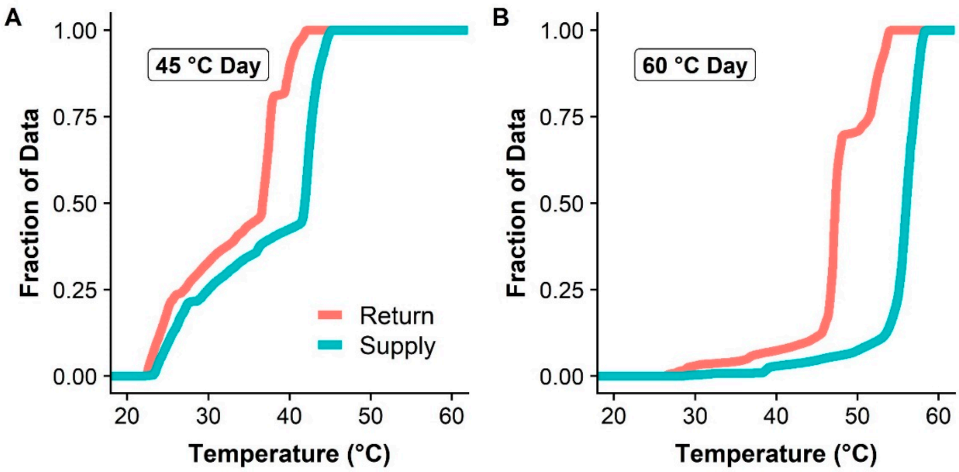

Figure S9. Empirical cumulative distribution function of the hot water supply and return on the closest floor to the boiler (B Floor) on a day the boiler set point was A) 45 °C and B) 60 °C.

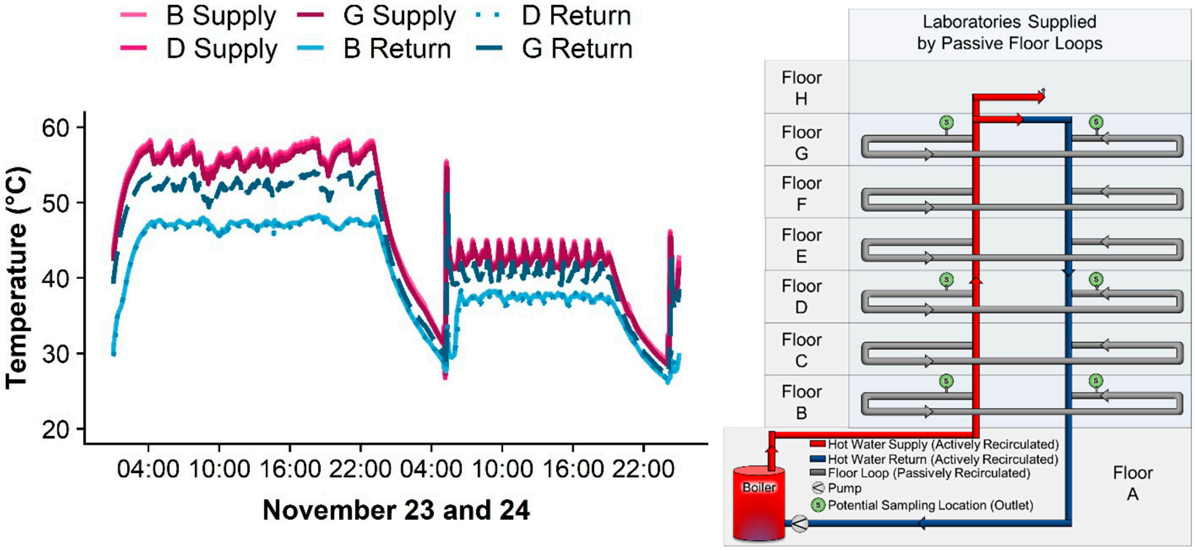

Figure S10. Pipe surface temperatures of the hot water recirculation supply and return pipes (left) and location where temperature sensors were installed (right).

All supply temperatures approached the water heater setpoint on a 60 °C (November 23) and a 45 °C (November 24) day. Heat loss to the environment (ambient loss and from the passive floor loops) is evident starting with Floor G and getting progressively larger as water is returned to the boiler.

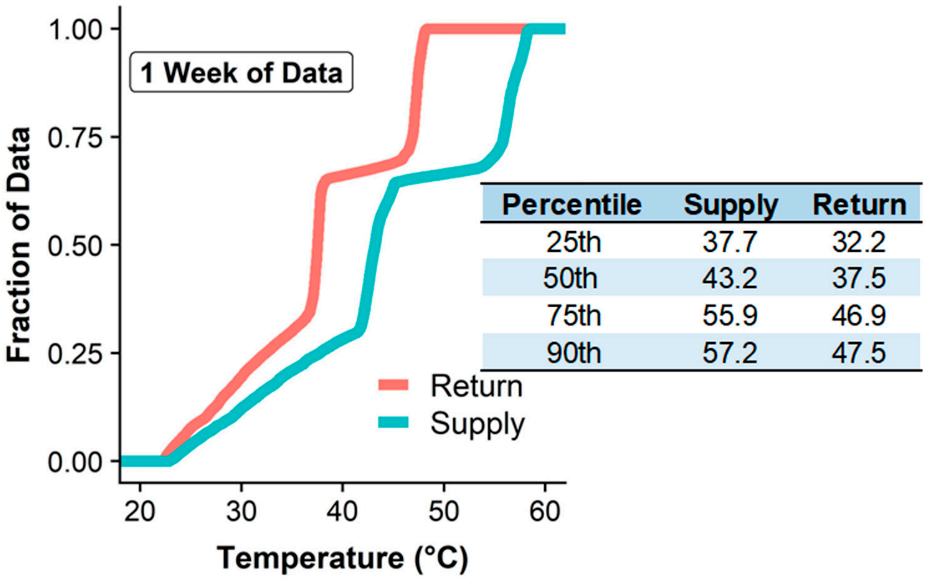

Percentile	Supply	Return
25th	37.7	32.2
50th	43.2	37.5
75th	55.9	46.9
90th	57.2	47.5

Figure S11. Empirical cumulative distribution function of the hot water supply and return on the floor closest to the boiler (B Floor) from 1 week of data.

The table reflects the 25th, 50th, 75th, and 90th percentile data collected

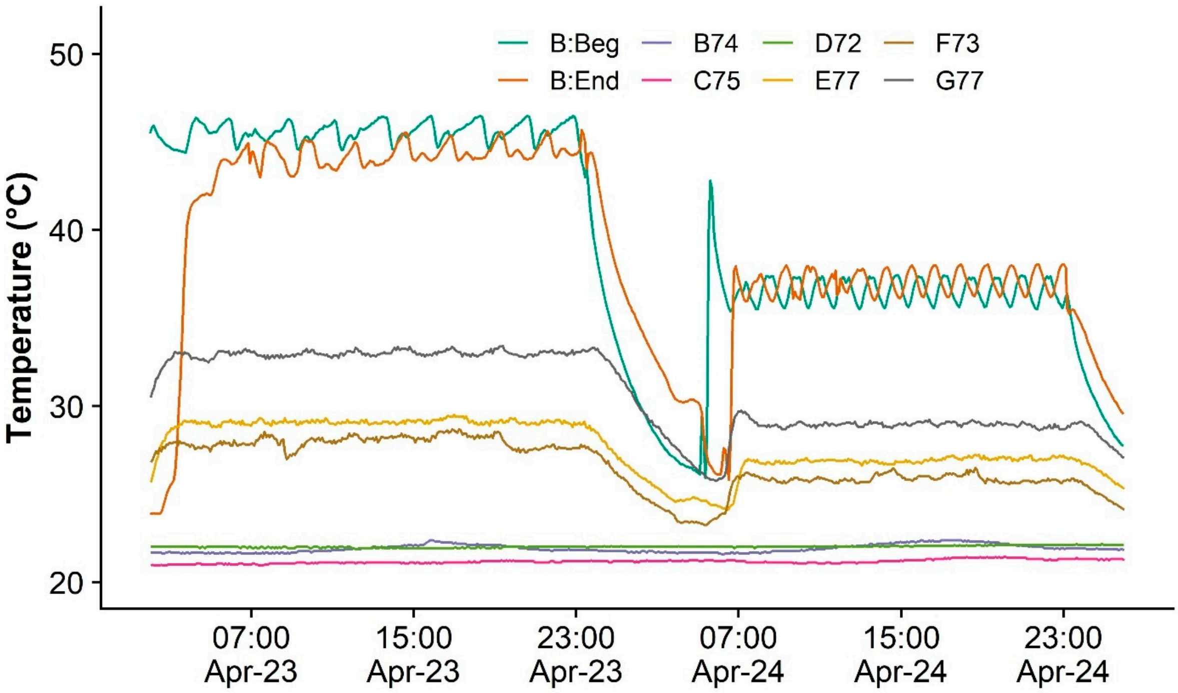

Figure S12. Pipe surface temperature from a typical mid-week period during the lockdown phase of data collection.

April 23, 2020 was a 60 °C boiler operation day; April 24, 2020 was a 45 °C boiler operation day. Data was collected at the inlet and outlet of the passive recirculating loops for Floor B and at the point where the vertical service pipe entered the rooms indicated on Floors B-G. Convective mixing between the passive floor loop and individual outlet is evident by the elevated temperature (relative to ambient) at the room supply pipe. Levels of exchange were partially related to the efficiency of their passive recirculation loop (increasing from B to G; with none on floor C due to a closed ball valve in the middle of the recirculating loop that prevented passive recirculation); however, there is also a large amount of variation based on the specific location and how well the sensor is attached to the pipe. Therefore, interpreting trends in passive recirculation is not warranted with the data collected.

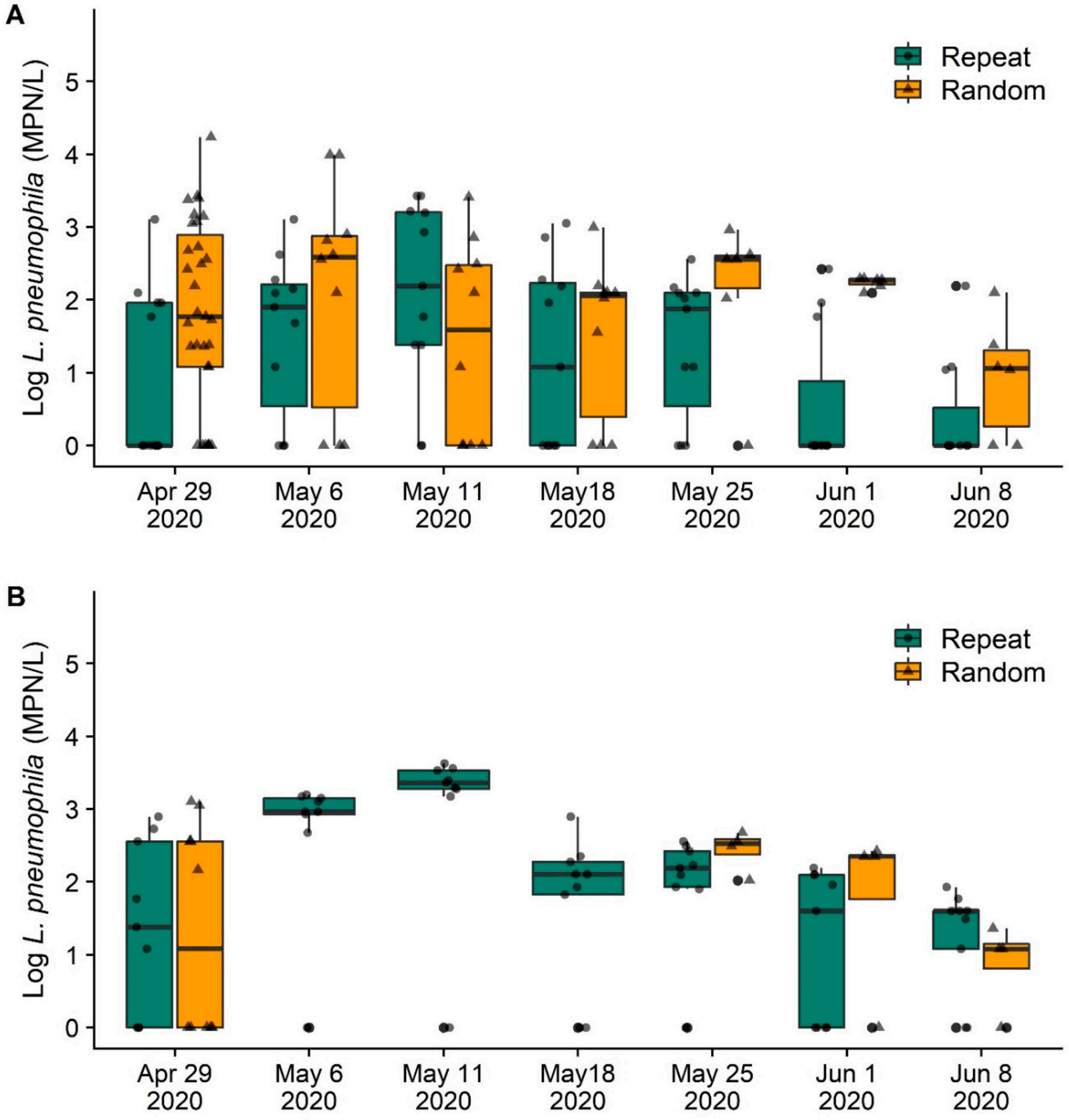

Figure S13. *L. pneumophila* culture numbers from immediately after the COVID Lockdown and during the Recommissioning phase of data collection between Repeatedly and Randomly sampled outlets for A) first draw samples and B) 5-minute flushed samples.

There was not a consistent significant difference in randomly compared to repeatedly sampled outlets. Note: no 5-minute flushed data were collected for random outlets on May 6, 11, or 18, 2020. L. pneumophila culture numbers may have decreased more rapidly in repeatedly sampled outlets relative to random outlets due to being exposed to elevated temperatures at least when sampling was conducted; however, there is not enough data to make concrete conclusions.

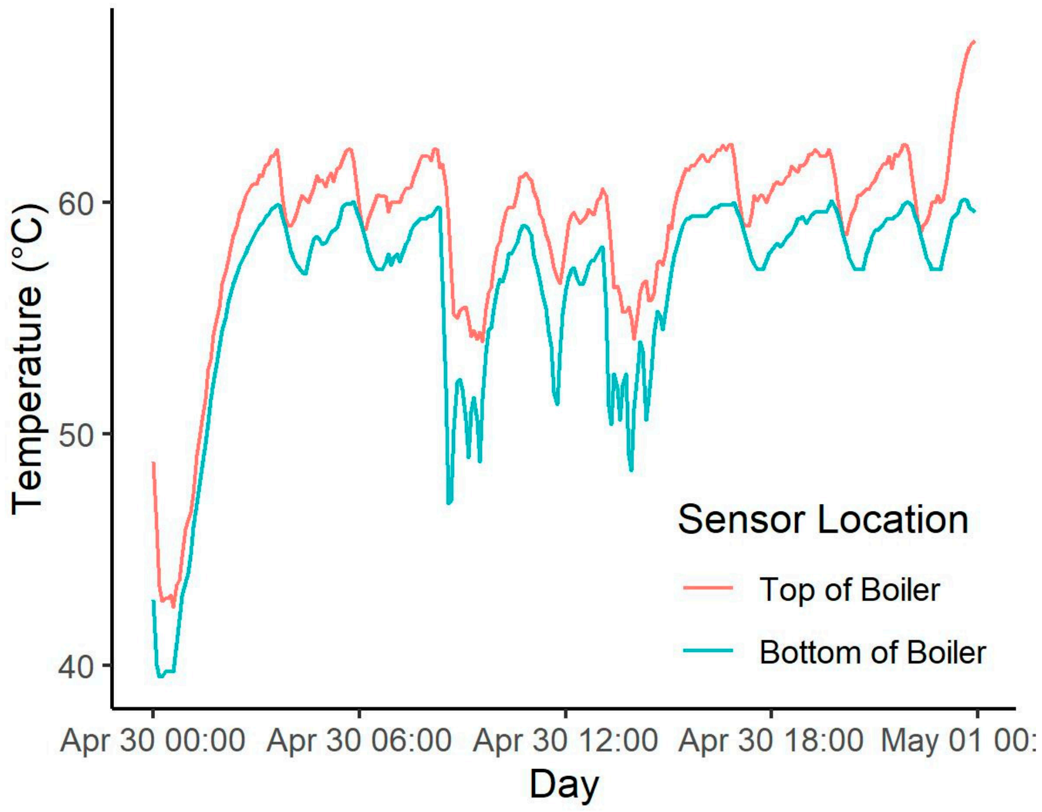

Figure S14. Water temperature at the boiler outlet ("Top of Boiler") and bottom of boiler during the recommissioning flushing activities on April 30, 2020. Recommissioning flushing began around 08:30 and ended around 16:00.

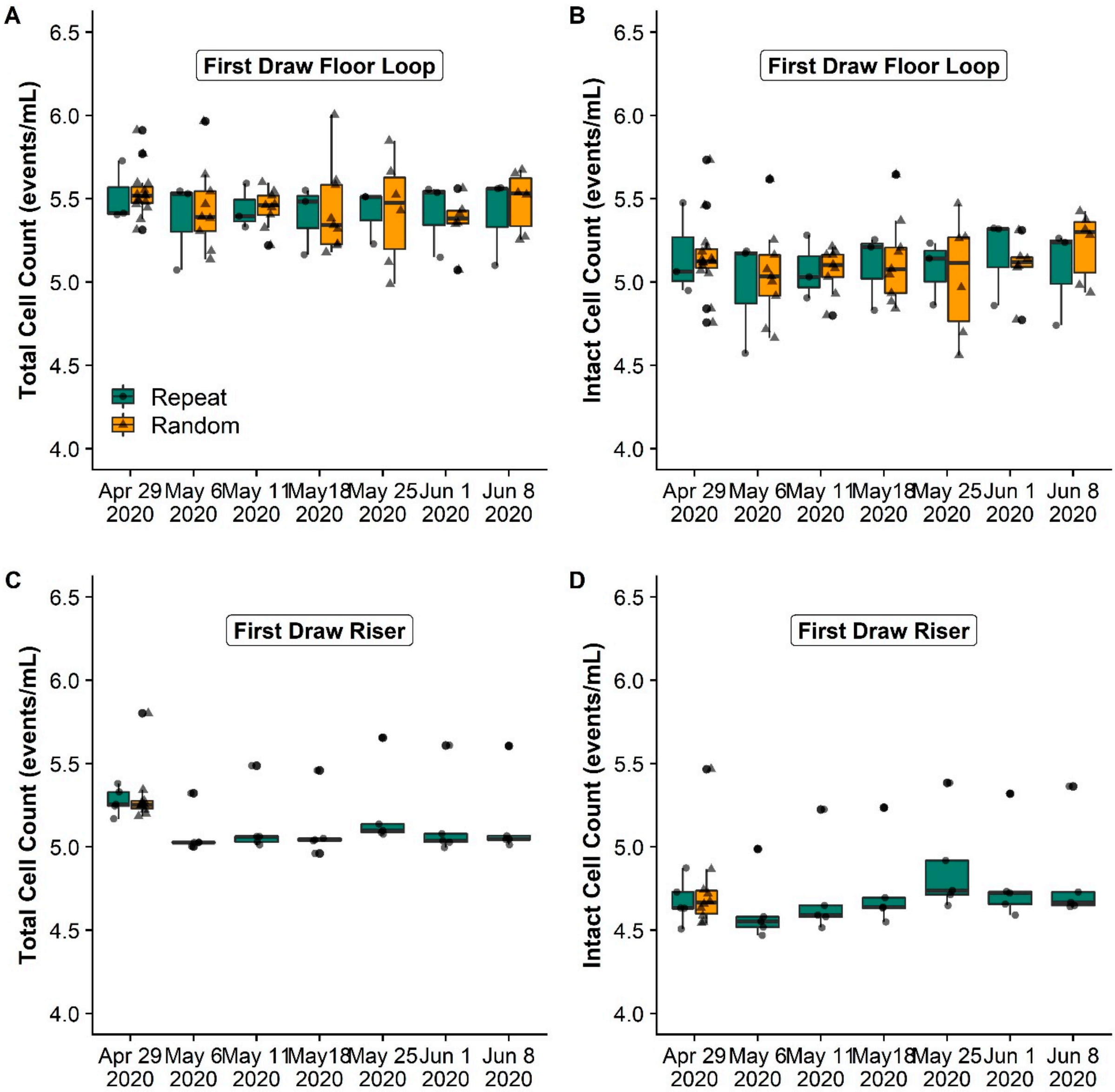

Figure S15. Total and intact cell counts for randomly compared to repeatedly sampled outlets in first draw samples from outlets served by the floor loops (panel A and B) and risers (panel C and D).

No significant differences between repeatedly and randomly sampled outlets was seen. Note: randomly sampled outlets were not conducted on riser outlets May 6, 2020 onward.

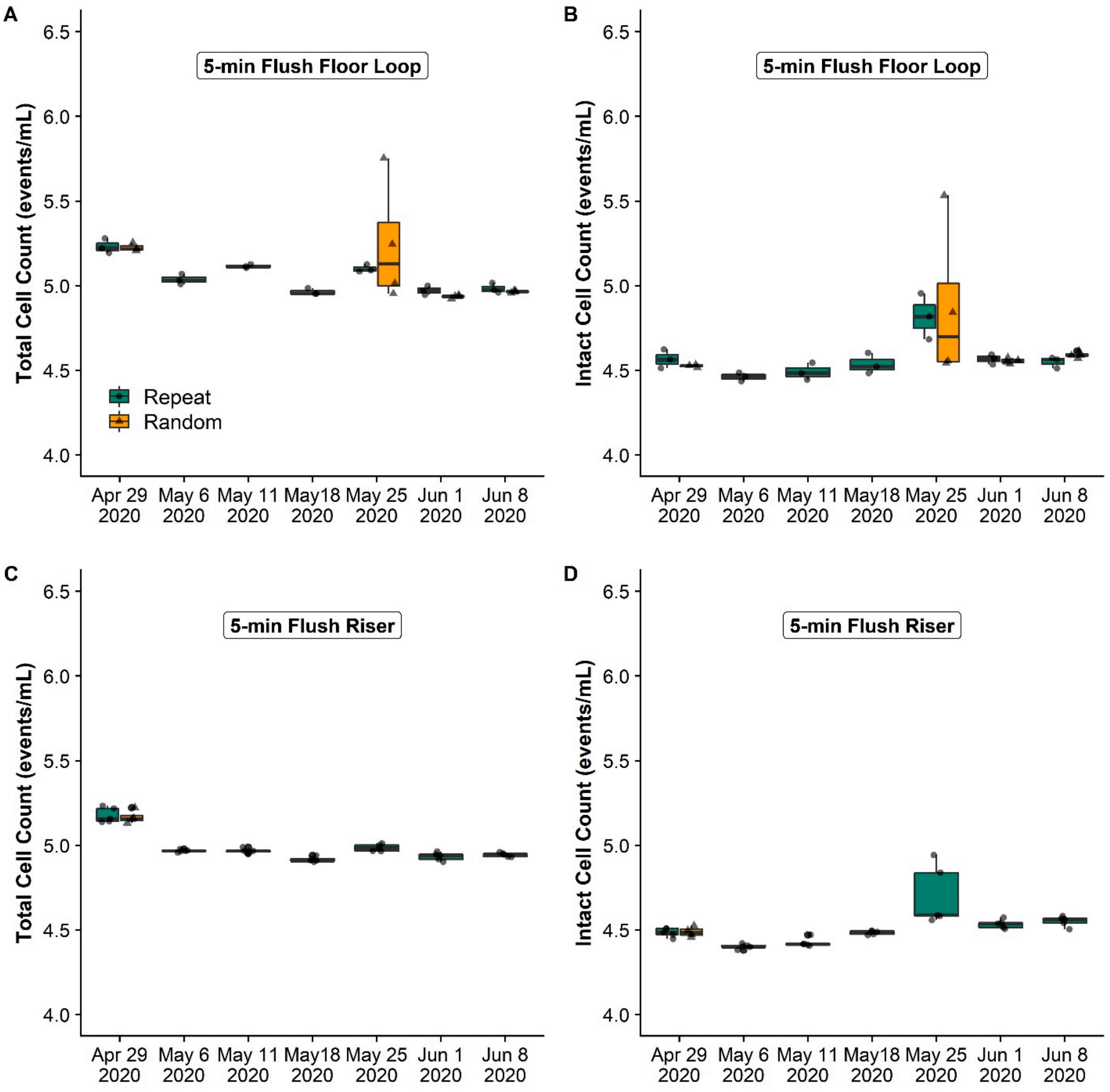

Figure S16. Total and intact cell counts for randomly compared to repeatedly sampled outlets in 5-minute flushed samples from outlets served by the floor loops (panel A and B) and risers (panel C and D).

No significant differences between repeatedly and randomly sampled outlets was seen. Note: randomly sampled outlets were not conducted on riser outlets May 6, 2020 onward.

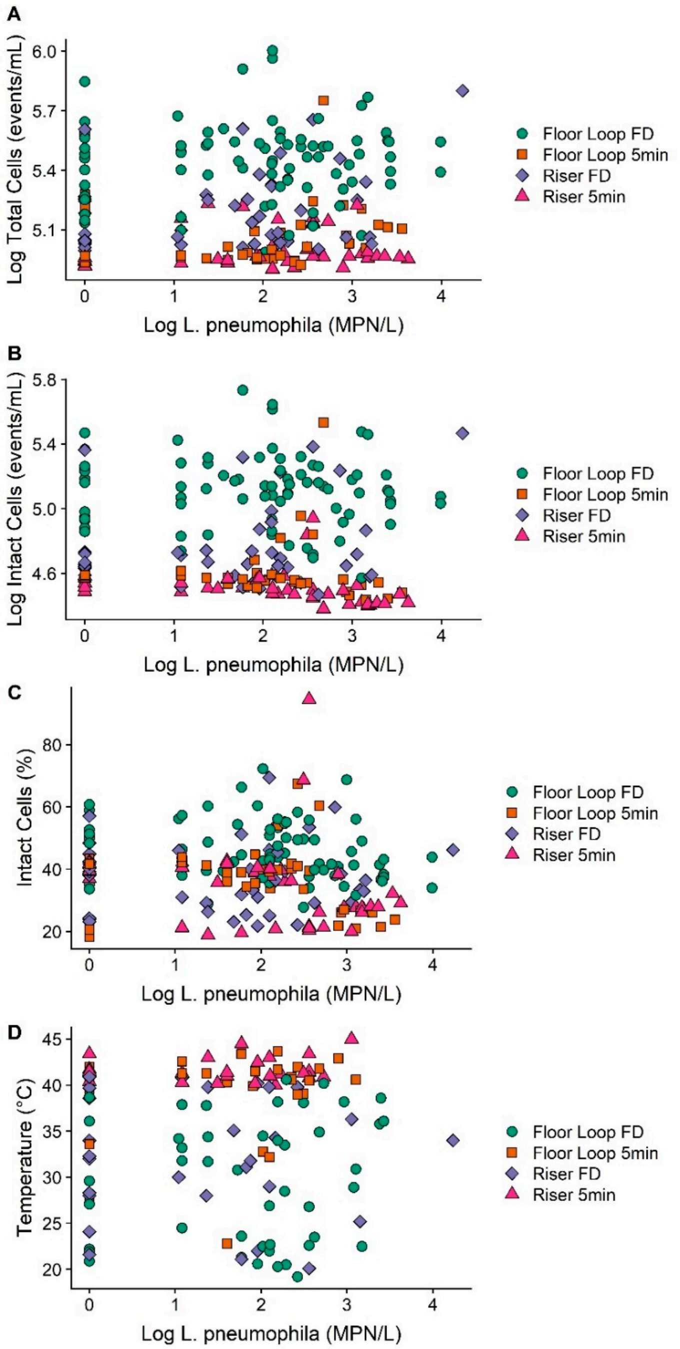

Figure S17. ***L. pneumophila*** **culture number trends with A) total cell counts, B) intact cell counts, C) percent of intact cell counts, and D) sample temperature. No discernable trends were identified.**
